# Supplementary figures and images for: Creating a general-purpose generative model for healthcare data based on multiple clinical studies
Source: PLOS Digit Health. 2025 Nov 5;4(11):e0001059. doi: 10.1371/journal.pdig.0001059 (PMC12588491; doi:10.1371/journal.pdig.0001059)

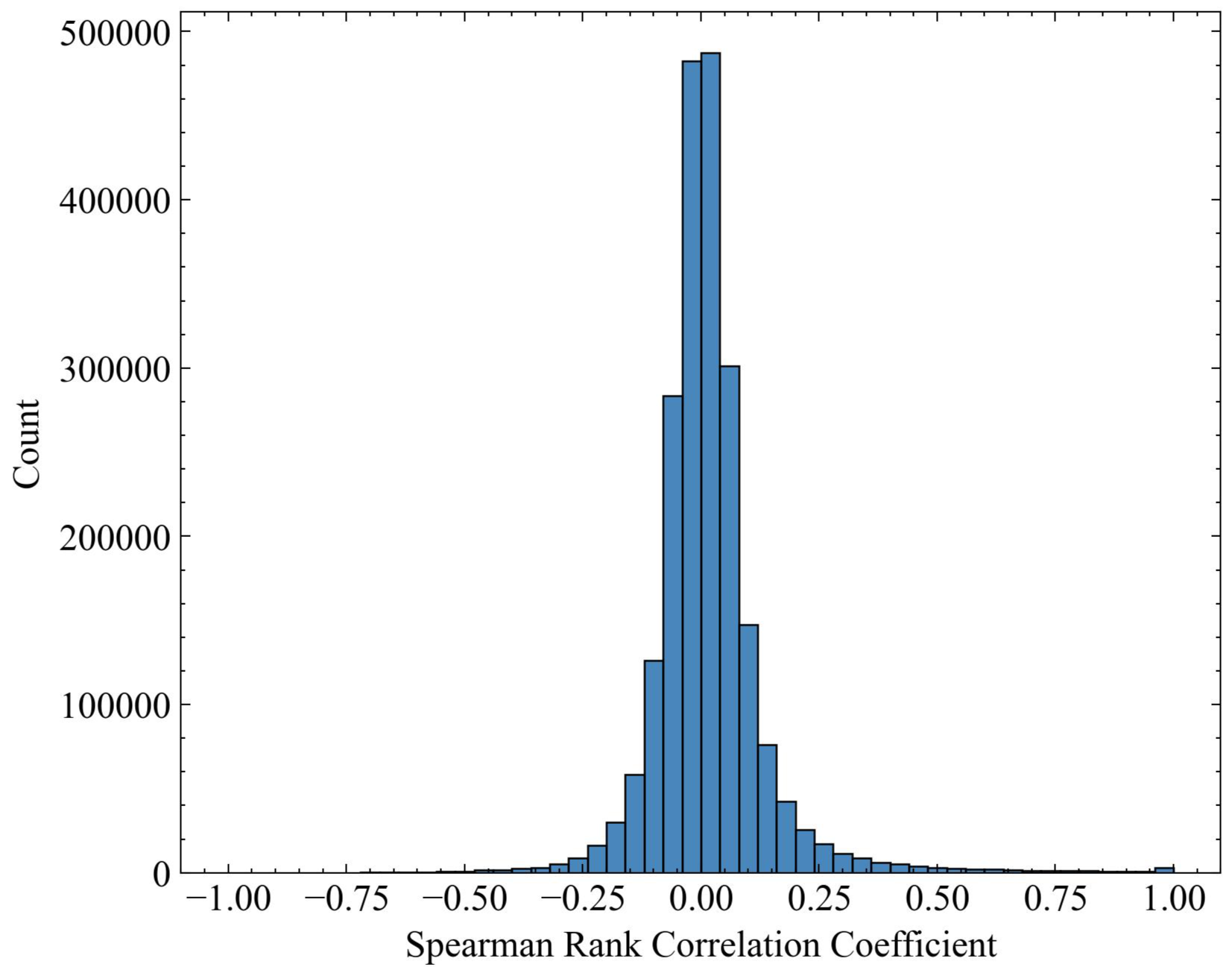

Supplement: S1 Fig — The number of combinations of selecting pairs of attributes from 1776 attributes is 1,088,550. For each combination, the Pearson rank correlation coefficient was obtained, and the histogram was created. (TIF) [file pdig.0001059.s001.tif]

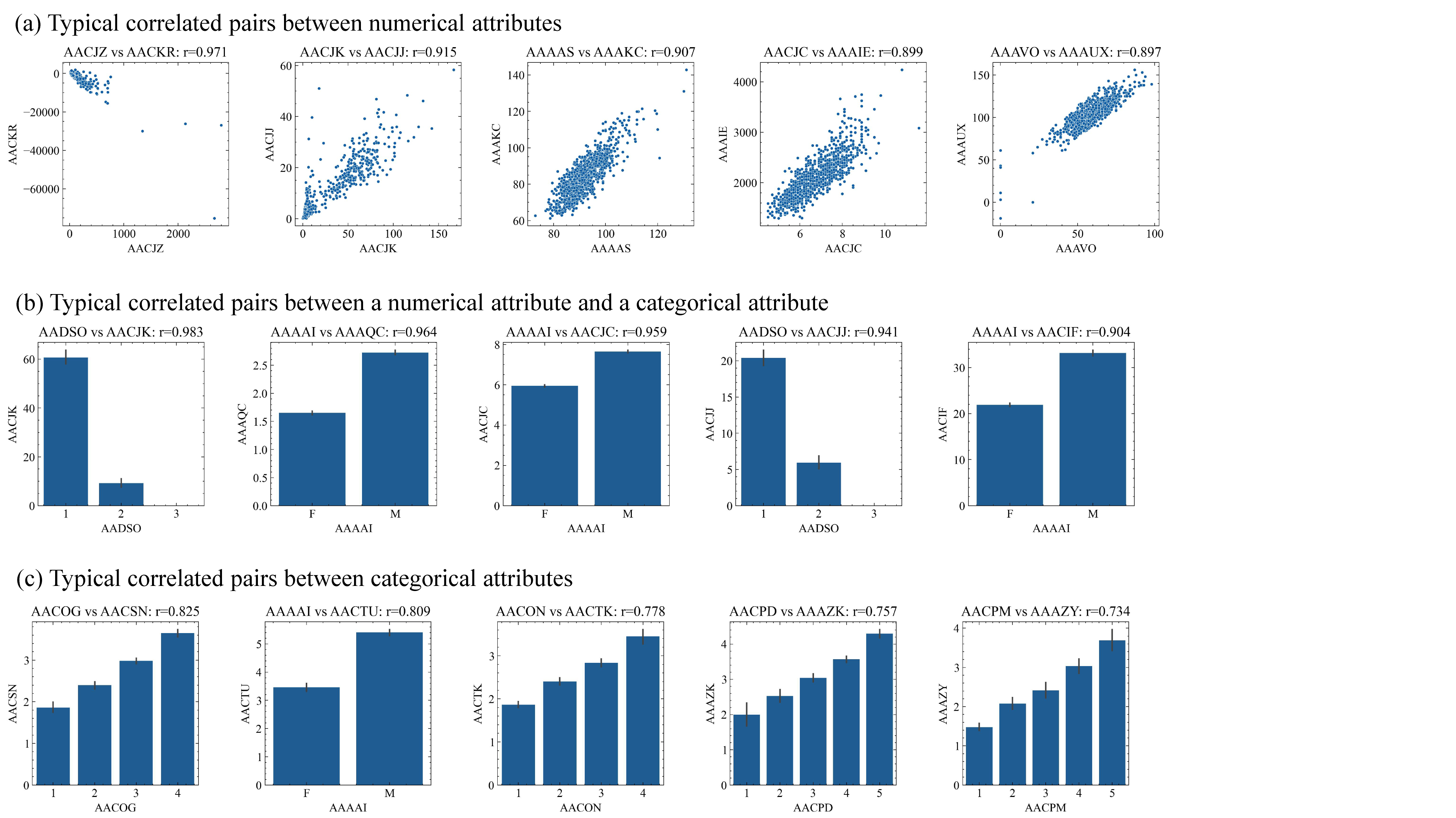

Supplement: S2 Fig — The ΦK correlation coefficient [40] was employed to assess the relationships between pairs of variables across various data types, including numerical, categorical, and ordinal. Each 5-letter code corresponds to an attribute definition. (a) Typical correlated pairs between numerical attributes. The numbers in the titles represent the correlation coefficient of the pairs. (b) Typical correlated pairs between a numerical attribute and a categorical attribute. The numbers in the titles represent the correlation coefficient of the pairs. (c) Typical correlated pairs between categorical attributes. The numbers in the titles represent the correlation coefficient of the pairs. (TIF) [file pdig.0001059.s002.tif]
