# Supplementary material for: Creating a general-purpose generative model for healthcare data based on multiple clinical studies
Source: PLOS Digit Health. 2025 Nov 5;4(11):e0001059. doi: 10.1371/journal.pdig.0001059 (PMC12588491; doi:10.1371/journal.pdig.0001059)
Supplement: Attachment — Submitted filename: PDIG_D_25_00094_Review.pdf [file pdig.0001059.s011.pdf]

Reviewer's Report for "Creating a General-Purpose Generative Model for Healthcare Data based on Multiple Clinical Studies"

May 22, 2025

Summary

This paper introduces the Virtual Human Generative Model (VHGM), a general-purpose generative model trained on multiple clinical datasets. The model integrates more than 2000 health attributes from various sources to support missing value imputation, synthetic data generation, and exploratory analysis. It is deployed as a commercial API and designed to support personalized and predictive health applications. While the concept is impactful and timely, the scientific rigor and clarity require significant improvement.

Major Comments

M1. Lack of External Validation

The model is only evaluated using internal hold-out sets. There is no validation using independent external datasets. This raises concerns about the model's generalizability beyond the original population.

Recommendation: Include evaluation on an external dataset such as NHANES or MIMIC to assess real-world applicability.

M2. Unclear Model Architecture

The paper references a prior publication for methodological details but does not describe the model architecture, learning algorithm, or hyperparameters sufficiently.

Recommendation: Provide a concise overview of the core architecture and algorithm within the paper for transparency and reproducibility.

M3. No Benchmark Comparison

There is no comparison with existing synthetic data generation models (e.g., CTGAN, medGAN). This makes it difficult to assess the performance advantage of VHGM.

Recommendation: Compare VHGM's performance on key tasks against at least one open-source baseline.

M4. Causal Inference Risk
The VHGM supports "what-if" queries, but the paper does not clearly differentiate between statistical associations and causal effects. This may lead to misinterpretation by end-users.

Recommendation: Add clearer caveats and limitations around causal interpretation of results.

M5. Limited Demographic Diversity and Bias Analysis
The training data is heavily Japan-centric. There is minimal discussion of how this bias impacts model utility for other populations.

Recommendation: Include a fairness or subgroup performance analysis and explicitly address the risks of population bias.

M6. Overemphasis on Deployment Without Scientific Benchmarking
The API deployment is discussed in detail, but the evaluation lacks standard scientific performance metrics (e.g., calibration error, predictive uncertainty, robustness).

Recommendation: Include quantitative performance metrics to establish scientific validity.

Minor Comments

m1. Terminology such as "scenario-based analysis" and "governance model" should be more precisely defined.

m2. Tables summarizing imputation error (e.g., Table 6–7) should include confidence intervals or standard deviations.

m3. Typographical issues exist in some figure captions; improve formatting consistency.

m4. While privacy is mentioned, no formal risk assessment is provided. Claims of privacy-preservation should be moderated unless supported by empirical analysis.

Overall Assessment

This paper presents a promising direction for general-purpose healthcare modeling and addresses a critical need in data-scarce settings. The VHGM is ambitious in scope and well-positioned for practical impact. However, the current

version falls short on scientific rigor due to a lack of external validation, architectural clarity, and performance benchmarking. With substantial revisions, the work has the potential to become a valuable contribution.

Recommendation

Major Revisions
