# Supplementary material for: Creating a general-purpose generative model for healthcare data based on multiple clinical studies
Source: PLOS Digit Health. 2025 Nov 5;4(11):e0001059. doi: 10.1371/journal.pdig.0001059 (PMC12588491; doi:10.1371/journal.pdig.0001059)
Supplement: Attachment — Submitted filename: Response to Reviewers.docx [file pdig.0001059.s012.docx]

Aug. 29, 2025

**Response to Reviewers**

Manuscript ID: PDIG-D-25-00094

Title: Creating a General-Purpose Generative Model for Healthcare Data based on Multiple Clinical Studies

To:

Dr. Leo Anthony Celi, Editor-in-Chief, PLOS Digital Health

Dr. Jasmit Shah, Guest Editor, PLOS Digital Health

We thank the Editor-in-Chief, Guest Editor, and the reviewers for their thoughtful and constructive feedback, which has helped us to improve the clarity, rigor, and overall quality of our manuscript.

**Revision Summary**

Below is a brief overview of the main changes made in response to the reviewers’ comments:

- Added external validation using the NHANES dataset and Japanese e-Stat dataset to evaluate model generalizability (Page 9, lines 294–309; Page 15, lines 465–473; Tables 8 and 9).
- Expanded Methods section to provide details on the model architecture and the training algorithm (Page 7, lines 219–249; Fig 3).
- Included benchmark comparisons with other generative model algorithms, including CTGAN, for synthetic data generation tasks (Page 9, lines 310–316; Page 15, lines 474–479; Table 10).

**Reviewer #1**

**Major Comments**

**M1. Lack of External Validation**

**Comment:** The model is only evaluated using internal hold-out sets. There is no validation using independent external datasets. This raises concerns about the model’s generalizability beyond the original population. Recommendation: Include evaluation on an external dataset such as NHANES or MIMIC to assess real-world applicability.

**Response:** We agree with the reviewer’s comment and have conducted an external validation analysis using two independent datasets: the National Health and Nutrition Survey of Japan (e-Stat) and the U.S. National Health and Nutrition Examination Survey (NHANES). For 24 nutritional intake attributes with high semantic equivalence across datasets, we calculated z-scores and overlap areas to assess statistical concordance between VHGM outputs and each external dataset. The results indicate greater concordance with e-Stat (mean overlap area = 0.82) than with NHANES (mean overlap area = 0.59), reflecting the Japanese origin of our training data. Details are provided in S4 Text.
**Location of changes in manuscript:** Page 9, lines 294–309; Page 15, lines 465–473; Tables 8 and 9

**M2. Unclear Model Architecture**

**Comment:** The paper references a prior publication for methodological details but does not describe the model architecture, learning algorithm, or hyperparameters sufficiently. Recommendation: Provide a concise overview of the core architecture and algorithm within the paper for transparency and reproducibility.

**Response:** We have expanded the Methods section to include a brief description of the model architecture and training algorithm. Specifically, we describe our transformer-based encoder-decoder architecture, handling of heterogeneous attribute types, masking strategy for missing value imputation, and a two-stage training procedure combining per-dataset and combined-dataset training. These additions are intended to improve transparency and reproducibility without requiring the reader to refer solely to the referenced work.
**Location of changes in manuscript:** Page 7, lines 219–249; Fig 3

**M3. No Benchmark Comparison**

**Comment:** There is no comparison with existing synthetic data generation models (e.g., CTGAN, medGAN). This makes it difficult to assess the performance advantage of VHGM. Recommendation: Compare VHGM’s performance on key tasks against at least one open-source baseline.

**Response:** We added a benchmark comparison against established generative models (TVAE, CTGAN, and Gaussian Copula) on a regression task involving 65 attributes related to exercise, nutrients, sleeping habits, stress, and tiredness. The results (Table 10) show that our method outperforms baseline models when using synthetic data alone and yields further improvements when synthetic data augment the original dataset.
**Location of changes in manuscript:** Page 9, lines 310–316; Page 15, lines 474–479; Table 10

**M4. Causal Inference Risk**

**Comment:** The VHGM supports “what-if” queries, but the paper does not clearly differentiate between statistical associations and causal effects. This may lead to misinterpretation by end-users. Recommendation: Add clearer caveats and limitations around causal interpretation of results.

**Response:** We have clarified that VHGM outputs reflect statistical associations, not causal effects. Additional caveats were added to the Discussion section to prevent misinterpretation, noting that “what-if” scenarios should not be taken as causal conclusions and are intended for exploratory purposes only. In addition, we corrected a typographical error on the word “causal”.
**Location of changes in manuscript:** Page 18, lines 548–551; Page 19, line 600

**M5. Limited Demographic Diversity and Bias Analysis**

Comment: The training data is heavily Japan-centric. There is minimal discussion of how this bias impacts model utility for other populations. Recommendation: Include a fairness or subgroup performance analysis and explicitly address the risks of population bias.

**Response:** We expanded the Discussion to address demographic bias from the external validation. The results confirm that model performance is higher for Japanese population–like datasets (e-Stat) than for demographically different datasets (NHANES), highlighting the limitation in generalizing to other populations.
**Location of changes in manuscript:** Page 18, lines 581–582

**M6. Overemphasis on Deployment Without Scientific Benchmarking**

Comment: The API deployment is discussed in detail, but the evaluation lacks standard scientific performance metrics (e.g., calibration error, predictive uncertainty, robustness). Recommendation: Include quantitative performance metrics to establish scientific validity.

**Response:** We agree that quality metrics such as precision, recall, and accuracy would be useful if the model were designed to give point estimates for a specific prediction task. The output of the VHGM is a probability distribution, for which these conventional metrics for point estimation do not apply. Moreover, the VHGM is general purpose, meaning that any combination of 2,000+ attributes can serve as input and any attribute can serve as output. Consequently, we cannot evaluate these metrics for all possible input-output combinations even if the model is used for point estimation. This is why we devised a multi-faceted metric consisting of imputation (point estimate) errors and univariate and bivariate (and scenario-based) analysis. We are fully aware that our evaluation approach is by no means comprehensive, and this remains a future challenge in our research. To make this explicit, we have revised the Limitations subsection of the manuscript to note the lack of comprehensiveness in our current evaluation framework.

As additional quality facets, we have added descriptions of the validation with external datasets and comparisons of machine-learning efficiency with other generative model algorithms.

Location of changes in manuscript: Pages 18–19, lines 584–590

**Minor Comments**

m1. Terminology such as “scenario-based analysis” and “governance model” should be more precisely defined.

**Response:** We appreciate the reviewer’s suggestion to provide clearer definitions for key terms. In the revised manuscript, we have incorporated explicit definitions for both “scenario-based analysis” and “governance process” to improve clarity and ensure consistent interpretation. In addition, we have expanded the Results section to detail the specific scenario examined in the scenario-based analysis and to clarify how the results were interpreted under these different conditions.

Location of changes in manuscript: Page 8, lines 285–288; Page 9, lines 322–325; Pages 14–15, lines 451–464

m2. Tables summarizing imputation error (e.g., Table 6–7) should include confidence intervals or standard deviations.

**Response:** We appreciate the reviewer’s suggestion to include measures of variability in Table 6. In accordance with this comment, we have added standard deviations alongside the mean values for all attribute types in the table. For Table 7, however, it is not appropriate to calculate standard deviations for the accuracy metric (“correct estimation rate”) when dealing with categorical type or ordered categorical type attributes. **Location of changes in manuscript:** Page 14, lines 433–435; Tables 6 and 7

m3. Typographical issues exist in some figure captions; improve formatting consistency.

**Response:** We reviewed and corrected figure captions for consistency in formatting.

**Location of changes in manuscript:** Multiple figure captions (Figs 1–9).

m4. While privacy is mentioned, no formal risk assessment is provided. Claims of privacy-preservation should be moderated unless supported by empirical analysis.

**Response:** We thank the reviewer for this comment. In the revised *Governance process* subsection, we now acknowledge that improved model inference performance may increase potential privacy risks. Following the committee’s recommendation, we conducted a preliminary assessment of membership inference attack vulnerability, which showed no immediate evidence of exploitable risk. We also note that a more comprehensive empirical evaluation is warranted and plan to address this in future work.

Location of changes in manuscript: Page 16, lines 487–495

**Reviewer #2**

**Comment:** The model was trained using data predominately from the Japanese population which significantly limits its external validity warranting a bit more discussion.
**Response:** We agree with the reviewer’s comment regarding the potential limitation in external validity due to the predominantly Japanese origin of our training data. As noted in our response to Reviewer #1, we conducted an external validation analysis using two independent datasets: the National Health and Nutrition Survey of Japan (e-Stat) and the U.S. National Health and Nutrition Examination Survey (NHANES). The results indicated greater concordance with e-Stat (mean overlap area = 0.82) than with NHANES (mean overlap area = 0.59), reflecting the demographic proximity of e-Stat to our training data. To address the reviewer’s request for more discussion, we have revised the Limitations section to explicitly state that the external dataset validation provides direct evidence of this limitation in generalizability.
**Location of changes in manuscript:** Page 9, lines 294-309; Page 15, lines 465–473; Tables 8 and 9; Page 18, lines 581–582

**Comment:** Some medical conditions are of course affected more than others by this limitation, but, as an example, infectious diseases endemic to areas outside of Japan would be an impossibility to address.
**Response:** We agree with the reviewer’s observation that the degree to which this limitation affects different medical conditions varies. In response, we have revised the *Limitations* subsection to explicitly note that certain medical conditions specific to populations or environments outside the scope of the training data may not be appropriately addressed. This general statement encompasses, for example, infectious diseases endemic to regions outside Japan, which are not represented in our training data.

**Location of changes in manuscript:** Page 18, lines 582–583

**Comment:** What was the rationale for threshold of 0.7 SD for imputation errors? Even this error threshold can have impacts on clinical outcomes and bias towards underrepresented groups, especially when the external validity is already impaired by sample selection.

**Response:** We thank the reviewer for this comment. In the original manuscript, the reference to “within 0.7” was not a predefined universal criterion but rather a summary of the observed imputation error in our evaluation. To avoid implying an arbitrary threshold and to increase transparency, we have revised the manuscript to report the actual mean and standard deviation of standardized errors for each attribute type in the holdout set, as shown in Tables 6 and 7. As also noted in our response to m2 of Reviewer #1, we have now included standard deviations alongside mean values for attribute types in the imputation error table, thereby making the variation in errors explicit. This change clarifies the range of imputation errors and avoids reliance on a single summary threshold, while enabling readers to better assess the practical implications for their specific application contexts. NOTE: The values in Table 6 and 7 have been updated to reflect the latest model update (See *the additional editorial changes* below)

**Location of changes in manuscript:** Pages 13–14, lines 430–443

**Comment:** The training algorithm is referenced, but some comment on the core architecture would strengthen the foundation quite a bit.

**Response:** We appreciate the reviewer’s suggestion. As noted in our response to M2 of Reviewer #1, we have expanded the Methods section to briefly outline the core architecture in addition to the training algorithm already described. The manuscript now summarizes our transformer-based encoder–decoder design, its handling of heterogeneous attribute types, and the masking strategy for missing value imputation, thereby providing a clearer foundation without relying solely on prior publications.

**Location of changes in manuscript:** Page 7, lines 219–249; Fig 3

**Comment:** What were some of the details about the process for consent to share data?

**Response:** We thank the reviewer for requesting clarification on the process for obtaining consent to share data. In addition to the existing description of informed consent for participation and anonymized data use, we have revised the *Ethics approval, informed consent, and participation* subsection to explicitly state that the consent form informed participants that statistical models developed through the use of their anonymized data may be used in the future by Kao Corporation or its commissioned contractors. This addition clarifies that participants were made aware of, and consented to, the potential future use of such models under the conditions described in the consent form.

**Location of changes in manuscript:** Page 3, lines 82–84

**Comment:** Some details about [the governance committee’s] composition would be helpful, including patient representatives, clinical expertise, ethicists, etc.

**Response:** We thank the reviewer for this comment. In the revised manuscript, we have expanded the description of the governance committee to clarify its composition. While the VHGM is designed primarily for preventive healthcare applications and therefore does not explicitly target patients, the committee membership is structured to ensure that users’ perspectives, healthcare demands, and ethical and privacy considerations are appropriately addressed. Specifically, we now state that the committee members are carefully nominated to cover diverse backgrounds, including life science researchers, clinical study experts, data scientists, and marketers with expertise in healthcare applications. This diversity ensures that relevant scientific, clinical, user, and ethical viewpoints are incorporated into the governance process.

**Location of changes in manuscript:** Page 10, lines 343–347

**Comment:** I do have significant concerns with the 'Health Asset' tool presented... the ethical nuances should be discussed in more detail... safeguards for the end user would strengthen the manuscript significantly.

**Response:** We thank the reviewer for raising these important ethical considerations regarding the “Health Asset” tool. We agree that the potential misuse of such information by third parties (e.g., insurance companies, employers, or governmental bodies) warrants careful attention, even if the intended use is to inform and empower individual users. In response, we have expanded the description of the application to clarify its scope, limitations, and safeguards, which are displayed on the website to inform end users. Specifically, we now explicitly state that the application is not a medical device and is not intended for the diagnosis, treatment, or prevention of any disease. We also note that the application indicates the results should be interpreted as general informative guidance rather than definitive medical or financial advice. This clarification is intended to prevent misunderstanding or misuse and to reinforce that the service is designed for voluntary, self-directed exploration by users to promote health-related behavior change, rather than for institutional decision-making or risk assessment. In addition, we explicitly prohibit discriminatory uses of the API under contractual terms with API users, and the multi-stakeholder governance committee continuously monitors API usage to ensure compliance as a further safeguard. These safeguards and their rationale have also been explicitly described in the Results section.
Location of changes in manuscript: Page 17, lines 528–541; Page 10, lines 347–349; Page 16, lines 496–502

**Comment:** Descriptions of other downstream tasks are lacking. If the model is fully intended for prediction tasks then quantifying this with precision, recall, global accuracy, etc would be helpful to read.

**Response:** As noted in our response to M6 of Reviewer #1, we have addressed this point there.

**Additional editorial changes**

In response to the reviewers’ requests, we updated S2 Text to revise the description of the imputation error calculation methods and added S4 Text to provide details of the external dataset.

In addition to the specific revisions requested by the reviewers, we incorporated updates to reflect the latest version of the model, which was released during the review process. Specifically, we revised Tables 3–5 and Fig 6, which present attribute characteristic summaries, as the updated model includes approximately 1% more attributes than the previous version. The model performance results in Tables 6–7 and Fig 7–9 were also updated accordingly. Furthermore, Table 11, which lists the published models, was revised. Minor related text edits and figure/table captions were also adjusted for consistency. These updates do not affect the discussions or conclusions of the study.

Additionally, we corrected minor inaccuracies and removed duplicate words in the manuscript.

We hope that our responses and revisions have adequately addressed the reviewers’ concerns, and we sincerely thank you again for your thoughtful and constructive feedback.

Sincerely,

**Hiroshi Maruyama**
Kao Corporation; Preferred Networks Inc.; The University of Tokyo
Email: maruyama@acm.org
